# TCA-EfficientSCI: A Lightweight Causal Baseline for Cross-Measurement Temporal Continuity in Snapshot Compressive Imaging

**DOI:** 10.3390/e28070742

**Published:** 2026-07-01

**Authors:** Mengyuan Liu, Xing Liu, Ziheng Cheng, Xin Yuan

**Affiliations:** 1 School of Engineering, Westlake University, Hangzhou 310030, China; liumengyuan@westlake.edu.cn (M.L.); chengziheng@westlake.edu.cn (Z.C.); 2 Zhejiang Key Laboratory of 3D Micro/Nano Fabrication and Characterization, Westlake Institute for Optoelectronics, Fuyang, Hangzhou 311421, China; liuxing31@wioe.westlake.edu.cn

**Keywords:** snapshot compressive imaging, coded aperture compressive temporal imaging, EfficientSCI, temporal continuity, video reconstruction, inverse problems, computational imaging

## Abstract

Snapshot compressive imaging (SCI), including coded aperture compressive temporal imaging (CACTI), reconstructs high-speed video frames from compressed low-frame-rate measurements. Most deep SCI reconstruction networks are designed around a measurement-wise formulation: each compressed exposure is reconstructed independently, and the resulting frame segments are concatenated to form a continuous video. This protocol is effective for within-measurement reconstruction, but it leaves cross-measurement temporal continuity largely unmodeled. Boundary artifacts such as flickering, texture drift, or motion jumps can therefore appear between adjacent reconstructed segments, even when frame-wise reconstruction metrics remain competitive. This work identifies and empirically analyzes the underexplored problem of cross-measurement temporal continuity in continuous SCI, and it provides TCA-EfficientSCI as a lightweight, causal, and reproducible baseline. The Temporal Context Adapter uses the last *m* reconstructed frames from the previous measurement as causal temporal context and injects this history through a gated residual feature pathway. A boundary consistency loss regularizes the predicted temporal variation across measurement boundaries without forcing adjacent frames to be identical. In a controlled three-seed comparison, Full TCA with boundary loss reduces mean Boundary Difference Error (BDE) by 2.23% relative to the matched-epoch EfficientSCI control while maintaining similar PSNR and SSIM. Correct-history inference gives BDE 0.01615, while zero and shuffled history give 0.01725 and 0.01810, respectively. The adapter adds 1,019,905 parameters, or 11.56% relative to the EfficientSCI baseline parameters, and it changes 256×256 mean latency from 54.35 ms to 68.58 ms per measurement in the profiling protocol. Rather than claiming broad reconstruction-quality improvement, this study highlights cross-measurement continuity as an important evaluation and design dimension for continuous SCI deployment.

## 1. Introduction

Snapshot compressive imaging (SCI) is a computational imaging technique that captures high-dimensional visual signals by compressing them into low-dimensional measurements during a single exposure. In video SCI, also referred to as coded aperture compressive temporal imaging (CACTI), multiple high-speed frames are modulated by time-varying coding masks and integrated into a single two-dimensional measurement [1,2]. SCI also includes spectral or hyperspectral snapshot compressive imaging, commonly represented by coded aperture snapshot spectral imaging (CASSI), where a three-dimensional spatial–spectral data cube is compressed into a two-dimensional measurement and computationally reconstructed [3]; recent deep reconstruction models such as HMDAU-Net illustrate this hyperspectral branch of SCI [4]. This acquisition paradigm shifts part of the burden from high-speed or high-dimensional sensing hardware to computational reconstruction, enabling high-speed video or hyperspectral recovery with reduced sensing bandwidth. The inverse problem is highly underdetermined, since a single measurement must explain multiple latent frames or spectral channels, and reconstruction therefore depends strongly on the prior encoded by the recovery algorithm.

SCI reconstruction has traditionally been formulated at the level of a single compressed measurement. Early model-based methods use iterative optimization with hand-crafted or externally supplied priors, including total variation (TV) regularization [5,6], sparsity-oriented optimization, and plug-and-play (PnP) denoising [7]. Deep unfolding and end-to-end networks later improved the practical efficiency of SCI reconstruction by learning data-driven spatio-temporal priors [8,9,10,11,12,13]. EfficientSCI [14], in particular, provides an effective and efficient backbone by combining dense connections with space-time factorization. These developments have mainly advanced within-measurement reconstruction, namely, the recovery of the *T* frames compressed within one exposure.

Continuous SCI acquisition, however, poses an additional requirement that is not fully captured by the usual single-measurement formulation. In practical CACTI systems, measurements are acquired sequentially, and the reconstructed frame segments must be concatenated into a continuous high-speed video. Most deep SCI networks process each measurement independently: given $y_{n}$ and its masks $M_{n}$, the network reconstructs only $X_{n}$, without explicit access to the temporal state produced by $X_{n-1}$ or ${\hat{X}}_{n-1}$. This measurement-wise paradigm leaves the transition between ${\hat{x}}_{n,T}$ and ${\hat{x}}_{n+1,1}$ weakly constrained. As a result, a reconstructed video may have competitive frame-wise PSNR or SSIM while still showing perceptually disturbing boundary artifacts, such as flickering, texture drift, or implausible motion changes at the junction between adjacent reconstructed segments.

Adjacent measurements in a continuous acquisition sequence are generated from temporally neighboring scene states and therefore contain exploitable temporal dependence. A measurement-wise reconstruction pipeline does not explicitly propagate this dependence across segment boundaries. In this work, BDE is used solely as an empirical boundary-error diagnostic; it is not an information-theoretic quantity or bound.

This distinction motivates a separate problem formulation: temporally continuous SCI reconstruction should consider both within-measurement reconstruction and cross-measurement temporal continuity. The latter is a deployment-level property of the reconstructed video sequence, rather than a conventional frame-wise fidelity objective. It is also naturally causal in online acquisition: when reconstructing the current measurement, future measurements may not be available, whereas the previous reconstruction is already known. A causal formulation is therefore appropriate for continuous SCI systems that reconstruct measurements as they arrive.

To empirically study this problem, we develop TCA-EfficientSCI as a lightweight causal baseline built on EfficientSCI rather than as a new high-capacity reconstruction backbone. For the *n*-th measurement, the last *m* reconstructed frames from the (n−1)-th measurement are used as historical context. Because these history frames are themselves reconstructed and may contain artifacts, we do not directly overwrite the current measurement features. Instead, we insert a Temporal Context Adapter (TCA) between the feature extraction module and the space-time reconstruction blocks of EfficientSCI. The adapter uses a gated residual fusion mechanism, allowing the network to modulate the influence of historical features while preserving the original EfficientSCI pathway.

We further introduce a boundary consistency loss and a boundary-oriented metric. The loss regularizes the temporal variation across adjacent measurement segments by matching the predicted boundary difference with the ground-truth temporal difference. This avoids forcing the last frame of one segment and the first frame of the next segment to be identical, which would suppress natural motion. For evaluation, Boundary Difference Error (BDE) complements PSNR and SSIM by directly measuring whether the reconstructed temporal change at measurement boundaries agrees with the reference sequence. The goal is not to replace standard reconstruction metrics, but to make the cross-measurement continuity dimension visible.

The main contributions of this work are therefore problem-oriented and baseline-oriented:

- Cross-measurement temporal continuity is treated as a separate issue in continuous CACTI reconstruction, beyond within-measurement frame recovery.
- Boundary behavior is made both measurable and trainable through BDE and a boundary consistency loss for adjacent measurement segments.
- TCA-EfficientSCI serves as a lightweight causal baseline using the last *m* reconstructed frames as context; experiments test it with several representative methods.

## 2. Related Work

### 2.1. Snapshot Compressive Imaging: Acquisition and Model-Based Reconstruction

CACTI encodes a temporal sequence of high-speed frames into a single two-dimensional measurement through coded aperture modulation and temporal integration [1,2]. CASSI belongs to the spectral or hyperspectral SCI branch, where coded apertures and spectral dispersion compress a spatial–spectral data cube into a two-dimensional measurement [3]. The video CACTI paradigm trades temporal resolution for spatial bandwidth, enabling high-speed video capture with low-frame-rate sensors. The resulting reconstruction problem is inherently ill-posed: recovering *T* frames from one measurement requires solving an underdetermined system that relies on image or video priors to regularize the solution space.

Model-based reconstruction methods address this ill-posedness through explicit prior modeling within iterative optimization frameworks. Total variation (TV) regularization and sparsity-oriented priors exploit spatial smoothness and signal structure [5,6]. Optimization algorithms such as generalized alternating projection (GAP) [5], ADMM [15], and other alternating-direction methods [16] provide efficient solvers for the resulting constrained optimization problems. Plug-and-play (PnP) methods extend this framework by replacing the proximal operator with a pre-trained deep denoiser, decoupling the data fidelity term from the prior model and enabling the use of expressive learned priors within classical optimization loops [7,17,18]. While these methods offer flexibility with respect to mask design and image dimensions, their iterative nature imposes a fundamental trade-off between reconstruction quality and computational cost, limiting their applicability to large-scale video reconstruction scenarios.

### 2.2. Deep Learning-Based SCI Reconstruction

Deep neural networks have largely supplanted iterative methods for SCI reconstruction by learning expressive spatio-temporal priors from data and performing fast feed-forward inference. The resurgence of deep learning, catalyzed by breakthrough architectures such as AlexNet [19] and ResNet [20], has transformed computational imaging by enabling end-to-end learning of complex inverse mappings. The development of deep SCI methods can be broadly categorized into three paradigms: deep unfolding, end-to-end architectures, and hybrid designs.

Deep unfolding methods bridge optimization-based and learning-based approaches by unrolling iterative algorithms into trainable network architectures. Early formulations such as LISTA [8], ADMM-Net [9], and ISTA-Net [10] established the principle of embedding optimization steps as network layers with learnable parameters. For SCI specifically, domain-specific unfolding architectures have been developed with enhanced priors, including dense deep unfolding with 3D-CNN priors [12] and dual prior unfolding [13]. These methods preserve the interpretability of optimization while learning data-driven priors, though they remain constrained by the underlying optimization structure.

End-to-end architectures bypass the optimization paradigm entirely and learn direct mappings from measurements to reconstructed frames. The Transformer architecture [21], originally proposed for natural language processing, has influenced many vision models through its self-attention mechanism for capturing long-range dependencies. Its adaptation to vision tasks, exemplified by the Vision Transformer (ViT) [22] and Swin Transformer [23], has shown useful capability in modeling global contextual relationships. In the SCI domain, BIRNAT [24] introduces bidirectional recurrent networks with adversarial training for video SCI, capturing temporal dependencies through recurrent propagation. STFormer [25] uses spatial-temporal Transformer modeling, while EfficientSCI [14] and its CNN-Transformer extension [26] provide efficient large-scale SCI reconstruction backbones.

Hybrid and efficient designs aim to balance reconstruction quality with computational efficiency. EfficientSCI [14] combines dense connections with space-time factorization to provide an efficient reconstruction backbone. Its extensions include hybrid CNN-Transformer architectures [26]. Motion-aware approaches explicitly model temporal dynamics through motion regularization [27,28], while domain-factorized methods exploit structured video priors [29]. Architectural innovations from the broader image restoration community, including residual denoising networks [30], multi-stage architectures [31], and efficient Transformers [32], have also informed SCI network design.

Despite these advances, many commonly used SCI reconstruction pipelines still operate in a measurement-wise manner: each compressed measurement is reconstructed independently, without explicit temporal state transfer across adjacent measurements. Table 1 summarizes representative and recent methods from the perspective of temporal modeling and cross-measurement state. Recent work on epipolar Transformers for light-field super-resolution and disparity estimation [33] is included as an example of structured-dependency modeling in computational imaging, but it is discussed only as related work rather than as a numerical CACTI/SCI baseline because its light-field forward model and evaluation protocol differ from SCI. This measurement-wise paradigm is the central problem analyzed in the present work.

### 2.3. Temporal Consistency in Video Reconstruction

Temporal consistency—the perceptual smoothness and physical plausibility of frame transitions—is a critical quality dimension in video restoration and reconstruction. Frame-level artifacts that may be imperceptible in isolated image evaluation become prominently visible when results are viewed as a continuous sequence. Several research directions have been developed to enforce temporal coherence in video tasks.

Recurrent propagation architectures propagate temporal information across frames through sequential processing. BasicVSR [34] establishes bidirectional recurrent propagation as a core design principle for video super-resolution, demonstrating that information flow across temporal neighbors is essential for coherent reconstruction. BasicVSR++ [35] extends this with second-order grid propagation and flow-guided deformable alignment. The recurrent video restoration Transformer (RVRT) [36] combines recurrent architectures with guided deformable attention and has shown useful temporal modeling ability across multiple video restoration tasks.

Motion-aware alignment methods explicitly model inter-frame motion to enable feature-level temporal alignment. The classical Lucas–Kanade algorithm [37] and variational approaches [38] laid the foundation for optical flow estimation. Modern learning-based optical flow estimators such as RAFT [39] provide dense motion fields that guide feature warping across frames. Task-oriented flow (TOFlow) [40] learns motion representations optimized for specific downstream tasks rather than using generic flow. Deformable convolution-based alignment [41,42] offers a more flexible alternative by learning spatial offsets for adaptive feature aggregation. These alignment strategies are particularly relevant to video frame interpolation [43,44] and video inpainting [45], where temporal coherence directly depends on accurate motion estimation.

Temporal modeling in Transformers exploits self-attention mechanisms to capture long-range temporal dependencies. Building upon the Transformer architecture [21] and its vision adaptations [22,23], video restoration Transformers (VRT) [46] introduce temporal mutual self-attention that jointly attends across spatial and temporal dimensions. These approaches complement recurrent methods by enabling non-local temporal interactions, though at higher computational cost.

While these methods have made considerable progress in enforcing temporal consistency within fully observed video sequences, the temporal discontinuity problem in SCI reconstruction is fundamentally different. In video restoration, all frames are directly observed (possibly degraded), and temporal consistency addresses frame-to-frame instability in the restoration process. In continuous SCI reconstruction, by contrast, adjacent reconstructed segments are often generated from separate compressed measurements with no explicit shared temporal state. The boundary inconsistency arises not from restoration artifacts alone but from the absence of cross-measurement context during reconstruction. This distinction motivates a causal baseline: rather than enforcing consistency through alignment or propagation within a single reconstruction, TCA-EfficientSCI uses previous reconstructions as historical context for the current measurement.

## 3. Materials and Methods

This section defines the CACTI/SCI measurement model, the EfficientSCI baseline, the Temporal Context Adapter, the boundary loss, and the causal inference procedure.

### 3.1. Measurement Model

Let $y_{n}\in R^{H\times W}$ denote the *n*-th compressed measurement captured by the SCI system, where *H* and *W* denote the spatial height and width, respectively. Let $X_{n}=\left\{x_{n,1},x_{n,2},\ldots ,x_{n,T}\right\}\in R^{T\times H\times W}$ denote the corresponding *T* high-speed video frames. The CACTI/SCI forward imaging model is formulated as

(1) $$y_{n}=\sum_{t=1}^{T}M_{n,t}⊙x_{n,t}+\eta_{n},$$

where $M_{n,t}\in R^{H\times W}$ denotes the binary coding mask applied to the *t*-th frame, ⊙ denotes the Hadamard product, and $\eta_{n}\in R^{H\times W}$ represents additive measurement noise. The set of coding masks associated with the *n*-th measurement is denoted as $M_{n}={\left\{M_{n,t}\right\}}_{t=1}^{T}$. The objective of SCI reconstruction is to recover the latent video frames $X_{n}$ from the compressed measurement $y_{n}$ and the corresponding masks $M_{n}$.

### 3.2. EfficientSCI Baseline

The EfficientSCI framework [14] addresses the SCI inverse problem through an end-to-end neural network architecture that combines dense connections with space-time factorization. In its standard formulation, each measurement is reconstructed independently:

(2) $${\hat{X}}_{n}=R_{\theta }\left(y_{n},M_{n}\right),$$

where $R_{\theta }$ denotes the reconstruction network parameterized by θ, and ${\hat{X}}_{n}$ represents the reconstructed frames. The network architecture consists of two main components: (1) a feature extraction module that forms a measurement-aware representation by incorporating the coding masks, and (2) space-time reconstruction blocks that recover the latent frames through spatio-temporal feature processing.

This formulation recovers the *T* frames within each measurement, but Equation (2) carries no state across measurements. The reconstruction of measurement *n* is independent of the preceding ground-truth frames $X_{n-1}$ and reconstructed frames ${\hat{X}}_{n-1}$. When reconstructed segments are concatenated, this missing cross-measurement context can produce boundary discontinuities.

### 3.3. Temporal Context Adapter Baseline

#### 3.3.1. Problem Formulation and Design Rationale

Measurement-wise reconstruction lacks an explicit scene state at the boundary between adjacent measurements. To build a reproducible causal baseline, we use temporal context from the preceding measurement. The last *m* reconstructed frames from measurement n−1 are selected as the history input:

(3) $${\hat{H}}_{n-1}=\left\{{\hat{x}}_{n-1,T-m+1},{\hat{x}}_{n-1,T-m+2},\ldots ,{\hat{x}}_{n-1,T}\right\},$$

where 1≤m≤T is the history length hyperparameter. The baseline reconstruction formulation augments EfficientSCI by conditioning on this history:

(4) $${\hat{X}}_{n}=R_{\theta }\left(y_{n},M_{n},{\hat{H}}_{n-1}\right).$$

The history frames ${\hat{H}}_{n-1}$ are reconstructed rather than observed, so they may contain artifacts. Direct concatenation or unconditional fusion can pass these artifacts into the current measurement features. The fusion mechanism is therefore adaptive, allowing the network to modulate the influence of history.

#### 3.3.2. Adapter Architecture

**Figure 1:**
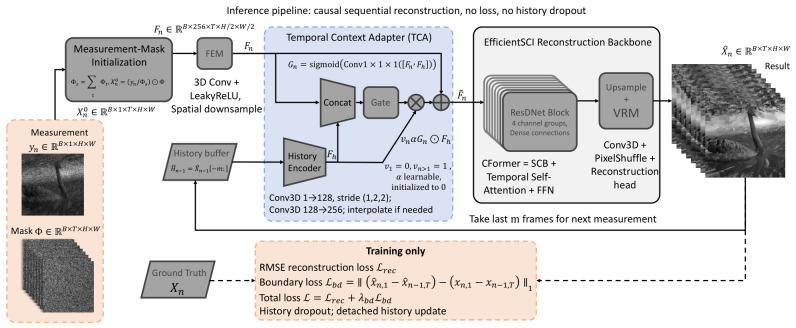
Architecture of TCA-EfficientSCI. Each compressed measurement is first processed by the EfficientSCI feature extraction module. For measurements after the first one, the last *m* reconstructed frames from the previous measurement are encoded as causal history and injected through the Temporal Context Adapter using gated residual fusion. The fused feature is then passed to the EfficientSCI space-time reconstruction blocks and reconstruction head. The first measurement uses an invalid-history flag, so the adapter is bypassed and the model follows the original EfficientSCI pathway.

We implement adaptive history fusion with a lightweight Temporal Context Adapter (TCA). Following adapter-based parameter-efficient learning [47,48,49,50], TCA is inserted between the EfficientSCI feature extraction module and the space-time reconstruction blocks. It preserves the baseline feature pathway while adding a small trainable history-fusion path. This simple design is intended as a reproducible reference point for continuous SCI methods. Figure 1 summarizes the causal reconstruction pipeline and the adapter position.

Let $F_{n}\in R^{B\times C\times T\times H^{\prime }\times W^{\prime }}$ denote the current measurement feature tensor extracted by the feature extraction module, where *B* is the batch size, *C* is the channel count, *T* is the number of reconstructed frames per measurement, and $H^{\prime },W^{\prime }$ denote the spatial feature dimensions. The history encoder $E_{h}$ processes the history frames to produce the encoded history feature:   

(5) $$F_{n}^{h}=E_{h}\left({\hat{H}}_{n-1}\right),$$

where $F_{n}^{h}\in R^{B\times C\times T\times H^{\prime }\times W^{\prime }}$ is aligned with $F_{n}$. The TCA is inserted after the 256-channel feature extraction module (FEM) output and before the ResDNetBlocks. The history encoder uses two Conv3D layers with LeakyReLU activations, mapping the grayscale history input through 1→128→256 channels. The first Conv3D layer uses stride (1,2,2) to match the FEM spatial resolution. The history feature is temporally aligned from *m* frames to *T* feature frames by trilinear interpolation with align_corners=False. The gate is a 1×1×1 Conv3D layer followed by a sigmoid activation.

The current measurement feature and the encoded history feature are fused through a gated residual mechanism. First, a gating signal is computed from the concatenated features:

(6) $$G_{n}=\sigma \left(\mathrm{Conv}3D_{1\times 1\times 1}\left(\left[F_{n},F_{n}^{h}\right]\right)\right),$$

where [·,·] denotes channel-wise concatenation, $\mathrm{Conv}3D_{1\times 1\times 1}$ reduces the concatenated channel dimension back to 256 channels, and σ(·) is the sigmoid activation function producing element-wise gating values in [0,1]. The fused feature is then computed as follows:

(7) $${\tilde{F}}_{n}=F_{n}+v_{n}\cdot \alpha \cdot G_{n}⊙F_{n}^{h},$$

where $v_{n}\in \left\{0,1\right\}$ indicates whether history is available, with $v_{1}=0$ for the first measurement and $v_{n}=1$ afterward. The scalar α∈R is an unconstrained learned signed history scale, and ⊙ denotes element-wise multiplication. We initialize α=0, so the adapter initially follows the EfficientSCI pathway and learns fusion gradually. The gate $G_{n}$ suppresses unreliable history or passes informative history, while the residual form preserves the baseline feature path.

### 3.4. Boundary Consistency Loss

The reconstruction loss used in our experiments supervises the fidelity of individual frames within each measurement using the root mean square error (RMSE):

(8) $$L_{\mathrm{rec}}=\sqrt{\frac{1}{THW}\sum_{t=1}^{T}{∥{\hat{x}}_{n,t}-x_{n,t}∥}_{2}^{2}},$$

where ${∥\cdot ∥}_{2}^{2}$ denotes the squared pixel-wise error summed over each frame. However, this loss does not address cross-measurement temporal consistency at measurement boundaries.

A straightforward approach to enforcing boundary consistency would be to minimize the difference between the last frame of the previous segment (${\hat{x}}_{n-1,T}$) and the first frame of the current segment (${\hat{x}}_{n,1}$). However, this direct frame-matching objective is inappropriate for dynamic scenes: in natural video, consecutive frames exhibit temporal motion, and the boundary frames of adjacent measurements are typically not identical. Enforcing frame equality would suppress legitimate scene dynamics and introduce artificial temporal smoothing.

To address this, we introduce a boundary consistency loss that supervises the temporal difference at measurement boundaries rather than the frame values directly:

(9) $$L_{\mathrm{bd}}=\frac{1}{HW}{∥\left({\hat{x}}_{n,1}-{\hat{x}}_{n-1,T}\right)-\left(x_{n,1}-x_{n-1,T}\right)∥}_{1}.$$

This formulation constrains the predicted boundary temporal difference to match the ground-truth temporal change, thereby penalizing abnormal discontinuities introduced by independent reconstruction while preserving natural scene motion across measurement boundaries. The $L_{1}$ term is averaged over all pixels, and all training and evaluation values are computed on images normalized to [0,1].

In the training implementation, the previous segment reconstruction ${\hat{x}}_{n-1,T}$ used in Equation (9) is not detached, so the boundary-loss gradient can reach both adjacent segment reconstructions within the same training sample. This is distinct from the history tensor passed to the adapter, which is detached before being reused as input to the next segment.

The complete training objective combines the reconstruction loss and the boundary consistency loss:

(10) $$L=L_{\mathrm{rec}}+\lambda_{\mathrm{bd}}L_{\mathrm{bd}},$$

where $\lambda_{\mathrm{bd}}>0$ is a hyperparameter balancing the contribution of boundary consistency. For the first measurement in each sequence (n=1), the boundary consistency loss is not applied, as no preceding measurement or reconstruction is available.

### 3.5. Inference Procedure

During inference, measurements are processed sequentially. For n=1, the history is initialized as ${\hat{H}}_{0}=0$ and $v_{1}=0$, so TCA is bypassed and the model follows EfficientSCI. For n≥2, the last *m* frames of the preceding reconstruction are stored as ${\hat{H}}_{n-1}$ and passed to TCA with $v_{n}=1$. This procedure uses only the current measurement and previous reconstruction, so it is causal.

Algorithm 1 summarizes the causal TCA training and inference procedure.
**Algorithm 1** Causal TCA training and inference1.For the first measurement in a sequence, set ${\hat{H}}_{0}=0$ and $v_{1}=0$.2.For measurement *n*, extract the EfficientSCI feature tensor $F_{n}$ from $y_{n}$ and $M_{n}$.3.Encode the stored history ${\hat{H}}_{n-1}$ with the history encoder and temporally align it to length *T*.4.Compute the gate $G_{n}$ by Equation (6) and the fused feature ${\tilde{F}}_{n}$ by Equation (7).5.Reconstruct ${\hat{X}}_{n}$ with the EfficientSCI reconstruction blocks and head.6.During training, compute the reconstruction loss and, when n>1, the boundary loss in Equation (9).7.Store ${\hat{H}}_{n}={\hat{X}}_{n}\left[:,-m:\right]$ with gradient detach and set $v_{n+1}=1$. During training, independently sample a history-dropout mask for each history-using segment and multiply it with $v_{n+1}$.8.During inference, process measurements sequentially with no future measurements.

## 4. Experiments

The experiments are designed as an empirical analysis of temporally continuous SCI reconstruction. We do not position TCA-EfficientSCI as a new backbone for maximizing frame-wise PSNR. Instead, the central question is whether a lightweight causal and reproducible baseline can expose and partially address boundary-oriented temporal continuity while preserving the reconstruction fidelity of the EfficientSCI baseline.

### 4.1. Experimental Setup

We evaluate TCA-EfficientSCI on simulated SCI data generated from high-speed video sequences using Equation (1). When applicable, TCA-EfficientSCI is initialized from EfficientSCI and fine-tuned under the controlled setting below. The principal comparison uses three seeds for the matched-epoch EfficientSCI control, Full TCA without boundary loss, and Full TCA with boundary loss; structural ablations use seed 0 for component attribution.

Training uses DAVIS 2017 480p, yielding 4858 samples. Evaluation uses six grayscale 256×256 CACTI simulation datasets with T=8: Kobe (32 frames, K=4), Traffic (48 frames, K=6), Crash (32 frames, K=4), Aerial (32 frames, K=4), Drop (8 frames, K=1), and Runner (8 frames, K=1).

We also include a no-reference real fan pilot with T=8, 50 compressed 512×512 measurements, and 400 reconstructed frames. The measurement and grayscale mask images are converted to MAT-file Version 7.3 files containing fields meas and mask. Because this sequence does not provide ground-truth high-speed frames, we do not report real-data PSNR, SSIM, or ground-truth BDE.

Unless stated otherwise, training uses T=8, history length m=2, sequential length K=2, 128×128 crops, batch size 1, AdamW with weight decay $10^{-4}$, learning rates $10^{-4}$ for TCA and $10^{-5}$ for the EfficientSCI backbone, $\lambda_{\mathrm{bd}}=0.05$, history dropout 0.2, AMP, history detach, and gradient clipping at 1.0. Reconstructed history frames are detached before reuse as adapter inputs; boundary-loss gradients can still reach both adjacent segment reconstructions, as described in Section 3.4.

Fine-tuning has three stages: 30 epochs for the temporal module, 15 epochs for the temporal module plus resdnet_list.4–resdnet_list.7, and 5 epochs for the full network. No-history EfficientSCI controls use temporal_variant="none"; under the actual trainability selector, they train the full EfficientSCI backbone in all three stages. Thus the EfficientSCI control is matched to TCA variants in epoch count, data exposure, initialization, optimizer family, and evaluation protocol, but not in trainable parameters or parameter-update operations. It is used as a conservative matched-epoch control.

### 4.2. Evaluation Metrics

We use PSNR and SSIM for frame-wise reconstruction fidelity, and we report BPSNR and BSSIM for boundary-region fidelity when available. BPSNR and BSSIM denote PSNR and SSIM computed on the boundary-adjacent four-frame window for each valid measurement boundary, using the last two frames of segment *n* and the first two frames of segment n+1 and their corresponding ground-truth frames, and then averaged over valid boundary rows. To evaluate cross-measurement temporal continuity, we additionally use Boundary Difference Error (BDE). For a sequence with K>1 measurements, BDE is defined as

(11) $$\mathrm{BDE}=\frac{1}{K-1}\sum_{n=1}^{K-1}\frac{1}{HW}{∥\left({\hat{x}}_{n+1,1}-{\hat{x}}_{n,T}\right)-\left(x_{n+1,1}-x_{n,T}\right)∥}_{1}.$$

BDE measures the mean absolute error between the predicted and ground-truth temporal changes at measurement boundaries, with pixels normalized to [0,1]; lower values indicate better boundary consistency. For single-measurement sequences such as Drop and Runner, BDE is undefined because no cross-measurement boundary exists, so these sequences are excluded from boundary-average BDE calculations.

For real measurements without ground truth, PSNR, SSIM, and BDE cannot be computed. We therefore report no-reference diagnostics: boundary absolute difference, interior absolute difference, their ratio, mean temporal absolute difference, and mean Laplacian variance as a simple sharpness proxy.

### 4.3. Comparison with the EfficientSCI Baseline

The main comparison is designed to answer two conservative questions: (1) whether causal temporal context changes boundary-average BDE on sequences with valid cross-measurement boundaries, and (2) whether this boundary-oriented baseline preserves frame-wise reconstruction fidelity as measured by PSNR and SSIM. Table 2 reports the three-seed quantitative comparison with the matched-epoch EfficientSCI control.

The numerical changes in Table 2 are modest and should be interpreted accordingly. Full TCA with boundary loss changes the mean PSNR from 36.4437 dB to 36.4801 dB and the mean SSIM from 0.97491 to 0.97507 relative to the matched-epoch EfficientSCI control, while changing mean BDE from 0.01652 to 0.01615. The BDE relative change is −2.23%, and the paired sequence-cluster bootstrap interval for the BDE difference is [−0.000574, −0.000197] (Table 3). Full TCA without boundary loss also gives a lower mean BDE than the matched-epoch EfficientSCI control (0.01635 vs. 0.01652). These results do not suggest a large reconstruction-quality gain; rather, they show a lower mean boundary diagnostic under the controlled protocol while leaving the average frame-wise metrics nearly unchanged.

The history controls in Table 4 further test whether the adapter uses sequence-specific causal history. The zero-history control keeps the history-valid flag set to $v_{n}=1$ after the first measurement but replaces the history tensor with zeros. The shuffled-history control uses a fixed bank of reconstructed histories from the correct-history pass, selects history from a different sequence, and preserves the same measurement index by using the stored history for segment n−1 when reconstructing segment *n*. Correct history gives BDE 0.01615, while zero history gives 0.01725 and shuffled wrong-sequence history gives 0.01810.

### 4.4. Additional Baseline Context

To place the causal baseline in context without mixing released checkpoints and newly fine-tuned models, Table 5 separates the original EfficientSCI checkpoint from the matched-epoch EfficientSCI control. Representative non-EfficientSCI methods are discussed in Table 1; they are not used to make uncontrolled claims about BDE unless compatible local outputs are available.

The matched-epoch control is the appropriate EfficientSCI baseline for the principal comparison because it uses the same epoch count, data exposure, initialization, optimizer family, and evaluation protocol as the TCA variants, while retaining the full-backbone training behavior described in Section 4.1.

### 4.5. Per-Dataset Boundary Analysis

Table 6 reports BDE on the four test sequences that contain at least two measurements. Drop and Runner are excluded from this table because each contains only one measurement and therefore has no cross-measurement boundary.

The per-dataset results show that the boundary-weighted mean BDE is lower for Full TCA with boundary loss than for the matched-epoch EfficientSCI control. At the sequence level, the largest downward changes appear on Aerial and Traffic, while Kobe and Crash show smaller changes. This sequence-level variation is important: it indicates that a fixed boundary regularization strategy does not yield uniformly large BDE changes across all motion patterns, and that future continuous SCI methods may need adaptive confidence estimation for historical context and boundary supervision.

### 4.6. Real-Measurement Case Study

Table 7 reports the no-reference diagnostics on the real fan sequence. All methods use the same saved real measurements and grayscale masks, with only method-required formatting, tiling, and normalization conversions. Since the real sequence used in this study does not provide synchronized high-speed ground truth, these diagnostics are used only as a pilot case study and not as real-data accuracy metrics.

The real-measurement diagnostics show a different behavior from the simulated ground-truth benchmark. EfficientSCI gives lower absolute temporal differences, suggesting a more conservative and smoother reconstruction. TCA-EfficientSCI has a higher absolute boundary difference, but its boundary-to-interior ratio is lower, changing from 1.707 to 1.518. GAP-TV and EfficientSCI with boundary smoothing have still lower ratios in this no-reference diagnostic, while BIRNAT has larger absolute temporal differences. These ratios do not imply ground-truth accuracy: a lower ratio may reflect either a less disproportionate boundary transition or a larger interior-difference denominator. Different methods also have different average brightness and contrast, so absolute temporal differences and Laplacian variance are not scale-invariant cross-method ranking metrics. Since these are no-reference diagnostics on a single real sequence, they should be interpreted as qualitative observations rather than a definitive real-data accuracy comparison.

### 4.7. Ablation Study

The ablation study evaluates the effect of boundary supervision, direct history concatenation, encoded history, gated fusion, and history validity. Table 8 uses seed 0 for structural attribution, while the three-seed comparison in Table 2 remains the principal uncertainty estimate.

The variants in Table 8 are defined by explicit source configurations. “Boundary loss only” keeps the EfficientSCI architecture with temporal_variant="none" and adds the boundary loss in Equation (9) with $\lambda_{\mathrm{bd}}=0.05$. “Direct history concatenation” resizes the raw reconstructed history tensor from B×m×H×W to a single-channel tensor aligned with $F_{n}$, concatenates it with $F_{n}$ along the channel dimension to form C+1 channels, projects it back to C=256 output channels with a 1×1×1 Conv3D layer, and applies the residual form ${\tilde{F}}_{n}=F_{n}+v_{n}\alpha P\left(\left[F_{n},h_{r}\right]\right)$. “History encoder + ungated addition” uses the same two-layer history encoder as Full TCA but removes the sigmoid gate, retaining the learned scalar α in ${\tilde{F}}_{n}=F_{n}+v_{n}\alpha F_{n}^{h}$. “Full TCA” uses the gated residual fusion in Equation (7).

The structural ablation shows that EfficientSCI with boundary loss only, direct history concatenation, and ungated encoded addition do not match the seed-0 BDE of the complete gated TCA with boundary loss. Direct raw-history concatenation gives BDE 0.01637, while history encoder with ungated addition gives 0.01626 and Full TCA with boundary loss gives 0.01616 in this seed. Because this table uses one seed, it is used for mechanism attribution only.

### 4.8. Qualitative Analysis

**Figure 2:**
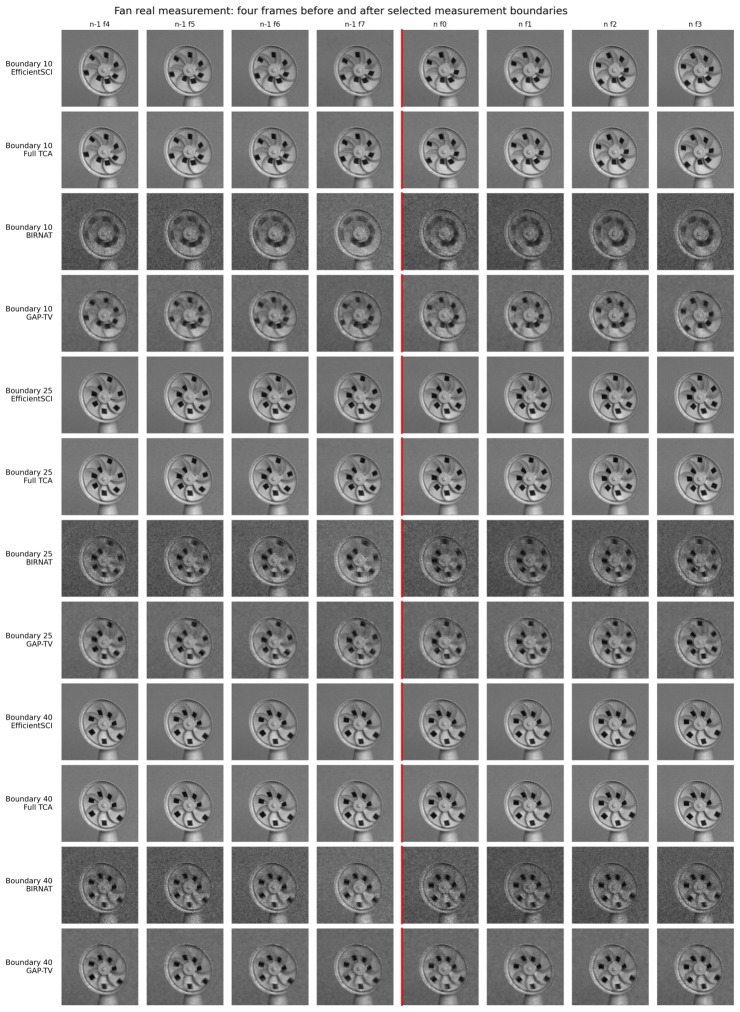
Real fan eight-frame boundary panels around selected measurement boundaries. Each panel contains the last four reconstructed frames from measurement *n* and the first four reconstructed frames from measurement n+1; the red vertical line marks the cross-measurement boundary. The figure is a qualitative no-reference diagnostic and is not a ground-truth error visualization.

**Figure 3:**
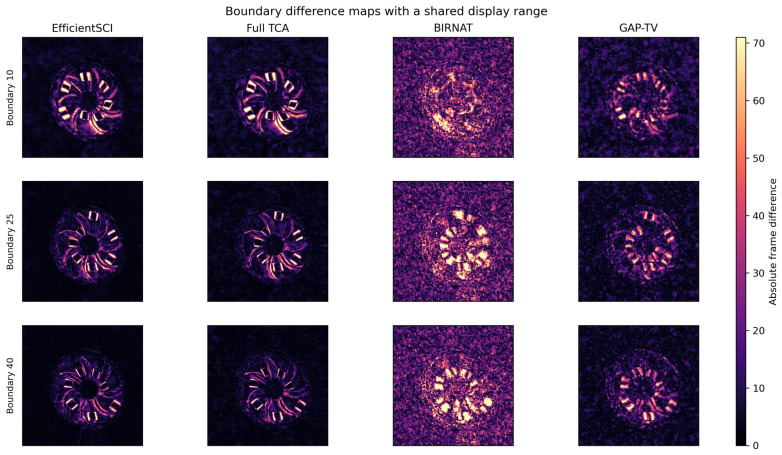
Boundary difference maps on the real fan sequence. These maps visualize temporal changes in saved reconstructions and are not ground-truth errors.

The qualitative evaluation is designed to inspect cross-measurement temporal consistency visually. Figure 2 shows selected eight-frame boundary panels from the real fan sequence. Each panel spans the last four frames reconstructed from measurement *n* and the first four frames reconstructed from measurement n+1. The red vertical line marks the cross-measurement boundary between the two groups of four frames. Figure 3 further visualizes temporal difference maps around selected boundaries.

Because no ground-truth high-speed frames are available for this real measurement, Figure 2 and Figure 3 are intended as visual diagnostics rather than claims of quantitative accuracy. The multi-boundary view complements the no-reference temporal diagnostics in Table 7 by showing reconstructed motion before and after several measurement boundaries in a directly comparable form.

### 4.9. Computational Overhead

The additional overhead of TCA-EfficientSCI comes from the history encoder and gated fusion operation. Table 9 reports total parameters, counted MACs/FLOPs, full latency per compressed measurement, reconstructed frames per second, peak allocated GPU memory, and persistent history-buffer memory under the same profiling protocol. The profiling record was generated on an NVIDIA GeForce RTX 4090 using PyTorch 2.2.2+cu118, CUDA 11.8, cuDNN 8700, and driver 560.31.02, with batch size 1, T=8, and AMP enabled. For each model and resolution, the script runs 30 unmeasured warm-up calls, then 100 timed first-measurement calls and 100 timed subsequent-measurement calls. Timing uses time.perf_counter() with torch.cuda.synchronize() before and after each timed call. The single latency column is the arithmetic mean over the 200 timed calls, equivalently the equal-weight average of the first-measurement and subsequent-measurement means because each group has 100 calls. The table displays peak allocated GPU memory from torch.cuda.max_memory_allocated() after resetting CUDA peak-memory statistics before the timed section. MACs count Conv3D and Linear operations only; unsupported element-wise operations, interpolation, PixelShuffle, reshape/concat, division, and memory movement are excluded from the count, so FLOPs are reported as 2× counted MACs rather than as a full hardware operation trace.

The adapter adds 1,019,905 parameters over 8,824,321 EfficientSCI baseline parameters. This is 11.56% when the denominator is the baseline parameter count and 10.36% when the denominator is the new total parameter count; the two ratios are distinct. Although counted MACs increase by only about 3% at 256×256, per-measurement latency increases by roughly 26%. This is expected: the history encoder operates on full-resolution history frames, and the fusion path adds temporal interpolation, concatenation, the sigmoid gate, and extra memory movement—operations excluded from the counted Conv3D/Linear MACs but still contributing to wall-clock time and kernel-launch overhead. “Lightweight” here refers to parameter count and design simplicity rather than to negligible latency.

## 5. Discussion

The main message of this work is not that a small adapter produces a large improvement in conventional reconstruction metrics. Instead, this work identifies and empirically analyzes an underexplored problem: continuous SCI reconstruction contains a boundary-consistency dimension that can be partly decoupled from average frame-wise fidelity. The observed PSNR and SSIM changes are small, which is expected because these metrics are averaged over all reconstructed frames and are dominated by within-measurement reconstruction quality. Boundary artifacts, however, occur at a sparse set of temporal locations and may be perceptually salient when the reconstructed frames are viewed as a video. This makes boundary-oriented metrics useful as complementary diagnostics rather than replacements for PSNR and SSIM.

BDE is sensitive to a different failure mode from frame-wise PSNR, but the current changes are small and should not be over-interpreted. It evaluates whether the temporal change predicted at the junction between adjacent reconstructed segments agrees with the reference temporal change. A method can therefore maintain similar PSNR while altering BDE, as observed in Table 2. This separation is important for continuous deployment: a video sequence with high average frame fidelity may still contain visible boundary flicker if each segment is reconstructed independently. Conversely, a boundary regularizer may change abnormal transitions without necessarily improving every frame-wise metric. The current results should therefore be interpreted as preliminary evidence that cross-measurement temporal continuity deserves explicit evaluation, not as definitive proof that BDE alone captures perceptual temporal quality.

The lightweight causal design is also intentional. Offline bidirectional refinement may use future measurements and can potentially provide stronger temporal coherence, but it is less aligned with online CACTI acquisition, where measurements arrive sequentially. TCA-EfficientSCI uses only the previous reconstruction, making it compatible with causal reconstruction pipelines. Initializing the adapter scale α to zero and using the validity flag $v_{n}$ also makes the first measurement and disabled-history case reduce to the EfficientSCI baseline. This behavior is valuable for a reproducible baseline extension of an existing reconstruction model, because it limits the risk of disrupting the baseline pathway.

At the same time, important limitations remain. The valid-boundary BDE change is modest in absolute magnitude, and the structural ablation is limited to one shared seed for mechanism attribution. The history input depends on the quality of previous reconstructions, so severe artifacts in the previous segment may provide misleading context and may accumulate over long sequences. Extending the real-data study to additional CACTI sequences was not possible within the present scope, and the real sequence used in this study does not provide synchronized high-speed ground truth. We therefore retain the single fan sequence as a pilot case study, explicitly avoid real-data accuracy conclusions, and leave broader real-data evaluation to future work. These limitations are consistent with the role of TCA-EfficientSCI as a simple causal baseline rather than a definitive solution. Future work should therefore focus on stronger boundary visual evidence, normalized BDE-style measures, confidence-aware history fusion, longer-sequence evaluation, direct comparison with recurrent or bidirectional video restoration strategies when controlled outputs are available, and joint optimization of coding masks and temporally continuous reconstruction. Diffusion-based plug-and-play priors [17,18] may also provide alternative routes for incorporating temporal priors.

## 6. Conclusions

This paper studied cross-measurement temporal continuity in continuous CACTI/SCI reconstruction. While existing measurement-wise methods recover frames within each compressed measurement, they do not explicitly model the transition between adjacent reconstructed segments. To address this issue, we built TCA-EfficientSCI as a lightweight causal baseline that injects the last *m* reconstructed frames from the previous measurement through a gated Temporal Context Adapter. We also introduced a boundary consistency loss and used BDE to evaluate boundary temporal variation. On the simulated CACTI sequences, TCA-EfficientSCI reduced the boundary-weighted mean BDE from 0.01652 to 0.01615 under the controlled three-seed protocol while maintaining similar PSNR and SSIM. The real fan sequence was used only as a no-reference pilot case study and does not establish real-data accuracy or superiority.

## Figures and Tables

**Table 1:** Representative and recent related work. “Evaluation status” distinguishes methods evaluated with local compatible outputs from the literature-only context.

Method (Year)	Paradigm/Temporal Modeling	State/Causality	Strengths	Limitations	Evaluation Status
GAP-TV [5] (2014)	Model-based GAP with TV prior	No cross-measurement state; measurement-wise	Mask-flexible classical reference	Slow; lower local metrics than EfficientSCI-family models	Locally evaluated
BIRNAT [24] (2020)	Recurrent SCI with bidirectional propagation	Implicit recurrent state; non-causal	Useful recurrent SCI checkpoint reference	Does not isolate causal cross-measurement state	Locally evaluated
EfficientSCI [14] (2023)	Feed-forward SCI with within-measurement space-time factorization	No explicit state; measurement-wise	Efficient high-quality backbone	No state carried between measurements	Locally evaluated
EfficientSCI extensions [26] (2024)	CNN/Transformer SCI with improved within-measurement modeling	Typically no explicit state; measurement-wise unless adapted	Strong efficient reconstruction design	No compatible boundary-level local outputs	Literature-only
Deep Motion Regularizer [27] (2024)	Motion-regularized SCI with motion prior or regularization	No verified causal history state; implementation-dependent	Targets temporal dynamics	No compatible checkpoint/output in this study	Literature-only
Motion-aware SCI [28] (2025)	Motion-aware SCI with explicit or learned motion modeling	No verified causal history state; implementation-dependent	Addresses motion cues	No compatible checkpoint/output in this study	Literature-only
Epipolar Transformer [33] (2026)	Light-field Transformer with epipolar spatial-angular dependency	Not an SCI state; non-causal light-field processing	Relevant structured-dependency modeling	Different forward model; no SCI numerical comparison	Literature-only

**Table 2:** Three-seed comparison with the matched-epoch EfficientSCI control on simulated SCI data. Values are the mean ± sample standard deviation over seeds 0, 1, and 2.

Method	PSNR	SSIM	BDE	BPSNR	BSSIM
EfficientSCI matched-epoch	36.4437 ± 0.0046	0.97491 ± 0.00003	0.01652 ± 0.00002	32.2480 ± 0.0069	0.96400 ± 0.00005
Full TCA w/o boundary loss	36.4804 ± 0.0014	0.97510 ± 0.00003	0.01635 ± 0.00002	32.3222 ± 0.0039	0.96446 ± 0.00004
Full TCA with boundary loss	36.4801 ± 0.0034	0.97507 ± 0.00004	0.01615 ± 0.00002	32.3271 ± 0.0052	0.96442 ± 0.00006

**Table 3:** Seed-level values and paired bootstrap confidence intervals supporting Table 2. Resampling is performed by sequence clustering over valid boundary units.

**Seed-Level Values.**						
**Method**	**Seed**	**PSNR**	**SSIM**	**BDE**	**BPSNR**	**BSSIM**
EfficientSCI matched-epoch	0	36.4465	0.97495	0.01652	32.2490	0.96404
EfficientSCI matched-epoch	1	36.4463	0.97490	0.01649	32.2542	0.96402
EfficientSCI matched-epoch	2	36.4384	0.97488	0.01653	32.2406	0.96394
Full TCA w/o boundary loss	0	36.4820	0.97512	0.01635	32.3177	0.96447
Full TCA w/o boundary loss	1	36.4801	0.97511	0.01633	32.3249	0.96450
Full TCA w/o boundary loss	2	36.4792	0.97507	0.01636	32.3241	0.96442
Full TCA with boundary loss	0	36.4829	0.97509	0.01616	32.3222	0.96444
Full TCA with boundary loss	1	36.4809	0.97508	0.01613	32.3327	0.96446
Full TCA with boundary loss	2	36.4763	0.97503	0.01616	32.3265	0.96435
**Paired Bootstrap BDE Intervals.**						
**Comparison**	**Mean BDE Diff.**		**95% Paired Bootstrap CI**		**Clusters/Units**	
Full TCA w/o boundary loss vs. EfficientSCI matched−epoch	−0.000170		[−0.000284, −0.000056]		4/42	
Full TCA with boundary loss vs. EfficientSCI matched−epoch	−0.000369		[−0.000574, −0.000197]		4/42	
Full TCA with boundary loss vs. Full TCA w/o boundary loss	−0.000199		[−0.000277, −0.000108]		4/42	

**Table 4:** Inference-only history control for the three Full TCA with boundary loss checkpoints. The zero and shuffled controls do not retrain weights.

History Mode	PSNR	SSIM	BDE
Correct	36.4801 ± 0.0034	0.97507 ± 0.00004	0.01615 ± 0.00002
Zero	36.4112 ± 0.0077	0.97434 ± 0.00008	0.01725 ± 0.00014
Shuffled	36.3797 ± 0.0079	0.97377 ± 0.00015	0.01810 ± 0.00022

**Table 5:** Original checkpoint versus matched-epoch EfficientSCI control. This table separates released-checkpoint evaluation from the controlled 50-epoch fine-tuning protocol.

Method	PSNR	SSIM	BDE	BPSNR	BSSIM
Original EfficientSCI checkpoint	36.3952	0.97489	0.01646	32.2717	0.96400
EfficientSCI matched-epoch	36.4465	0.97495	0.01652	32.2490	0.96404

**Table 6:** Per-dataset BDE on sequences with cross-measurement boundaries. Values are means over three seeds for each sequence.

Method	Kobe	Traffic	Crash	Aerial	Unweighted Avg.	Boundary-Weighted Avg.
EfficientSCI matched-epoch	0.01344	0.02104	0.01292	0.01564	0.01576	0.01652
Full TCA w/o boundary loss	0.01341	0.02084	0.01283	0.01529	0.01560	0.01635
Full TCA with boundary loss	0.01335	0.02066	0.01261	0.01497	0.01540	0.01615

**Table 7:** No-reference temporal diagnostics on the real fan measurement sequence.

Method	Boundary AbsDiff	Interior AbsDiff	Ratio	Temporal AbsDiff	Lap. Var.	Mean Int.
EfficientSCI	0.02557	0.01498	1.707	0.01628	123.04	0.4687
TCA-EfficientSCI	0.02811	0.01852	1.518	0.01970	138.14	0.4863
BIRNAT	0.08451	0.03726	2.268	0.04306	137.48	0.3745
GAP-TV	0.03580	0.02691	1.330	0.02800	117.28	0.4287
EfficientSCI + boundary smoothing	0.01990	0.01535	1.297	0.01591	120.10	0.4683

The real fan sequence contains 50 measurements and 400 reconstructed frames at 512×512 resolution. Because no ground-truth high-speed frames are available, PSNR, SSIM, BPSNR, BSSIM, and BDE are not reported for this sequence. Mean Int. denotes the mean reconstructed intensity of the normalized frames.

**Table 8:** Seed-0 structural ablation. All rows use the same seed and are intended for mechanism attribution rather than seed-level uncertainty estimation.

Method	PSNR	SSIM	BDE	BPSNR	BSSIM	Params
EfficientSCI matched-epoch	36.4465	0.97495	0.01652	32.2490	0.96404	8,824,321
EfficientSCI + boundary loss only	36.4469	0.97492	0.01635	32.2484	0.96397	8,824,321
Direct history concatenation	36.4472	0.97487	0.01637	32.2471	0.96391	8,890,370
History encoder + ungated addition	36.4673	0.97500	0.01626	32.2944	0.96426	9,712,898
Full TCA w/o boundary loss	36.4820	0.97512	0.01635	32.3177	0.96447	9,844,226
Full TCA with boundary loss	36.4829	0.97509	0.01616	32.3222	0.96444	9,844,226

**Table 9:** AMP inference profiling under the same measurement setting. Latency is measured per compressed measurement; FPS is reconstructed frames per second with T=8.

Model	Res.	Params	MACs ($10^{12}$)	FLOPs ($10^{12}$)	Latency (ms)	FPS	Peak Allocated Mem. (MB)	Hist. Mem. (MB)
EfficientSCI	256 × 256	8,824,321	1.423	2.846	54.35	147.20	1075.1	0.0
EfficientSCI	512 × 512	8,824,321	5.693	11.385	293.64	27.24	4168.8	0.0
Full TCA	256 × 256	9,844,226	1.469	2.939	68.58	116.65	1079.0	0.5
Full TCA	512 × 512	9,844,226	5.878	11.756	350.76	22.81	4172.7	2.0

## Data Availability

The code, training configurations, evaluation scripts, source manifest, and machine-readable CSV/JSON result files that reproduce the reported tables will be made publicly available upon acceptance. The simulation data are derived from the publicly available DAVIS 2017 dataset.

## Abbreviations

The following abbreviations are used in this manuscript:

SCI	Snapshot compressive imaging
CACTI	Coded aperture compressive temporal imaging
CASSI	Coded aperture snapshot spectral imaging
TCA	Temporal context adapter
BDE	Boundary difference error
PSNR	Peak signal-to-noise ratio
SSIM	Structural similarity index measure

## References

[1] Llull P., Liao X., Yuan X., Yang J., Kittle D., Carin L., Sapiro G., Brady D.J. (2013). Coded Aperture Compressive Temporal Imaging. Opt. Express.

[2] Hitomi Y., Gu J., Gupta M., Mitsunaga T., Nayar S.K. Video from a Single Coded Exposure Photograph Using a Learned Over-Complete Dictionary. Proceedings of the IEEE/CVF International Conference on Computer Vision.

[3] Wagadarikar A.A., Pitsianis N.P., Sun X., Brady D.J. (2009). Video Rate Spectral Imaging Using a Coded Aperture Snapshot Spectral Imager. Opt. Express.

[4] Zheng S., Zhu M., Chen M. (2023). Hybrid Multi-Dimensional Attention U-Net for Hyperspectral Snapshot Compressive Imaging Reconstruction. Entropy.

[5] Liao X., Li H., Carin L. (2014). Generalized Alternating Projection for Weighted *ℓ*_2,1_ Minimization with Applications to Model-Based Compressive Sensing. SIAM J. Imaging Sci..

[6] Beck A., Teboulle M. (2009). A Fast Iterative Shrinkage-Thresholding Algorithm for Linear Inverse Problems. SIAM J. Imaging Sci..

[7] Venkatakrishnan S.V., Bouman C.A., Wohlberg B. Plug-and-Play priors for model based reconstruction. Proceedings of the IEEE Global Conference on Signal and Information Processing.

[8] Gregor K., LeCun Y. Learning Fast Approximations of Sparse Coding. Proceedings of the International Conference on Machine Learning.

[9] Yang Y., Sun J., Li H., Xu Z. Deep ADMM-Net for Compressive Sensing MRI. Proceedings of the Advances in Neural Information Processing Systems.

[10] Zhang J., Ghanem B. ISTA-Net: Interpretable Optimization-Inspired Deep Network for Image Compressive Sensing. Proceedings of the IEEE/CVF Conference on Computer Vision and Pattern Recognition.

[11] Monga V., Li Y., Eldar Y.C. (2021). Algorithm Unrolling: Interpretable, Efficient Deep Learning for Signal and Image Processing. IEEE Signal Process. Mag..

[12] Wu Z., Zhang J., Mou C. Dense Deep Unfolding Network With 3D-CNN Prior for Snapshot Compressive Imaging. Proceedings of the IEEE/CVF International Conference on Computer Vision (ICCV).

[13] Zhang J., Zeng H., Cao J., Chen Y., Yu D., Zhao Y.P. Dual Prior Unfolding for Snapshot Compressive Imaging. Proceedings of the IEEE/CVF Conference on Computer Vision and Pattern Recognition.

[14] Wang L., Cao M., Yuan X. EfficientSCI: Densely Connected Network with Space-Time Factorization for Large-Scale Video Snapshot Compressive Imaging. Proceedings of the IEEE/CVF Conference on Computer Vision and Pattern Recognition.

[15] Boyd S., Parikh N., Chu E., Peleato B., Eckstein J. (2011). Distributed Optimization and Statistical Learning via the Alternating Direction Method of Multipliers. Found. Trends Mach. Learn..

[16] Yang J., Zhang Y., Yin W. (2010). A Fast Alternating Direction Method for TVL1-L2 Signal Reconstruction from Partial Fourier Data. IEEE J. Sel. Top. Signal Process..

[17] Zhu Y., Zhang K., Liang J., Cao J., Wen B., Timofte R., Van Gool L. Denoising Diffusion Models for Plug-and-Play Image Restoration. Proceedings of the IEEE/CVF Conference on Computer Vision and Pattern Recognition Workshops.

[18] Hurault S., Kamilov U., Leclaire A., Papadakis N. (2023). Convergent Bregman Plug-and-Play Image Restoration for Poisson Inverse Problems. Advances in Neural Information Processing Systems.

[19] Krizhevsky A., Sutskever I., Hinton G.E. ImageNet Classification with Deep Convolutional Neural Networks. Proceedings of the Advances in Neural Information Processing Systems.

[20] He K., Zhang X., Ren S., Sun J. Deep Residual Learning for Image Recognition. Proceedings of the IEEE/CVF Conference on Computer Vision and Pattern Recognition.

[21] Vaswani A., Shazeer N., Parmar N., Uszkoreit J., Jones L., Gomez A.N., Kaiser L., Polosukhin I. Attention Is All You Need. Proceedings of the Advances in Neural Information Processing Systems.

[22] Dosovitskiy A., Beyer L., Kolesnikov A., Weissenborn D., Zhai X., Unterthiner T., Dehghani M., Minderer M., Heigold G., Gelly S. An Image is Worth 16x16 Words: Transformers for Image Recognition at Scale. Proceedings of the International Conference on Learning Representations.

[23] Liu Z., Lin Y., Cao Y., Hu H., Wei Y., Zhang Z., Lin S., Guo B. Swin Transformer: Hierarchical Vision Transformer using Shifted Windows. Proceedings of the IEEE/CVF International Conference on Computer Vision.

[24] Cheng Z., Lu R., Wang Z., Zhang H., Chen B., Meng Z., Yuan X. (2020). BIRNAT: Bidirectional Recurrent Neural Networks with Adversarial Training for Video Snapshot Compressive Imaging. Computer Vision—ECCV 2020.

[25] Wang L., Cao M., Zhong Y., Yuan X. (2022). Spatial-Temporal Transformer for Video Snapshot Compressive Imaging. arXiv.

[26] Cao M., Wang L., Zhu M., Yuan X. (2024). Hybrid CNN-Transformer Architecture for Efficient Large-Scale Video Snapshot Compressive Imaging. Int. J. Comput. Vis..

[27] Chen Z., Li R., Li Y., Feng Y., Hou X., Qian X. (2024). Deep Motion Regularizer for Video Snapshot Compressive Imaging. IEEE Trans. Comput. Imaging.

[28] Li Z., Wang P., Zhang H., Li Z., Yuan X. Motion-Aware Reconstruction for Video Snapshot Compressive Imaging. Proceedings of the IEEE International Conference on Image Processing.

[29] Miao Y.C., Zhao X.L., Wang J.L., Fu X., Wang Y. (2024). Snapshot Compressive Imaging Using Domain-Factorized Deep Video Prior. IEEE Trans. Comput. Imaging.

[30] Zhang K., Zuo W., Chen Y., Meng D., Zhang L. (2017). Beyond a Gaussian Denoiser: Residual Learning of Deep CNN for Image Denoising. IEEE Trans. Image Process..

[31] Zamir S.W., Arora A., Khan S., Hayat M., Khan F.S., Yang M.H., Shao L. Multi-Stage Progressive Image Restoration. Proceedings of the IEEE/CVF Conference on Computer Vision and Pattern Recognition.

[32] Zamir S.W., Arora A., Khan S., Hayat M., Khan F.S., Yang M.H. Restormer: Efficient Transformer for High-Resolution Image Restoration. Proceedings of the IEEE/CVF Conference on Computer Vision and Pattern Recognition.

[33] Liang Z., Wang Y., Wang L., Yang J., Guo Y., Liu L., Zhou S., An W. (2026). Diving Into Epipolar Transformers for Light Field Super-Resolution and Disparity Estimation. IEEE Trans. Pattern Anal. Mach. Intell..

[34] Chan K.C.K., Wang X., Yu K., Dong C., Loy C.C. BasicVSR: The Search for Essential Components in Video Super-Resolution and Beyond. Proceedings of the IEEE/CVF Conference on Computer Vision and Pattern Recognition.

[35] Chan K.C.K., Zhou S., Xu X., Loy C.C. BasicVSR++: Improving Video Super-Resolution with Enhanced Propagation and Alignment. Proceedings of the IEEE/CVF Conference on Computer Vision and Pattern Recognition.

[36] Liang J., Fan Y., Xiang X., Ranjan R., Ilg E., Green S., Cao J., Zhang K., Timofte R., Van Gool L. Recurrent Video Restoration Transformer with Guided Deformable Attention. Proceedings of the Advances in Neural Information Processing Systems.

[37] Lucas B.D., Kanade T. An Iterative Image Registration Technique with an Application to Stereo Vision. Proceedings of the 7th International Joint Conference on Artificial Intelligence.

[38] Horn B.K.P., Schunck B.G. (1981). Determining Optical Flow. Artif. Intell..

[39] Teed Z., Deng J. RAFT: Recurrent All-Pairs Field Transforms for Optical Flow. Proceedings of the European Conference on Computer Vision.

[40] Xue T., Chen B., Wu J., Wei D., Freeman W.T. (2019). Video Enhancement with Task-Oriented Flow. Int. J. Comput. Vis..

[41] Tian Y., Zhang Y., Fu Y., Xu C. TDAN: Temporally-Deformable Alignment Network for Video Super-Resolution. Proceedings of the IEEE/CVF Conference on Computer Vision and Pattern Recognition.

[42] Zhu X., Hu H., Lin S., Dai J. Deformable ConvNets v2: More Deformable, Better Results. Proceedings of the IEEE/CVF Conference on Computer Vision and Pattern Recognition.

[43] Li Z., Zhu Z.L., Han L.H., Hou Q., Guo C.L., Cheng M.M. AMT: All-Pairs Multi-Field Transforms for Efficient Frame Interpolation. Proceedings of the IEEE/CVF Conference on Computer Vision and Pattern Recognition.

[44] Youk G., Oh J., Kim M. FMA-Net: Flow-Guided Dynamic Filtering and Iterative Feature Refinement with Multi-Attention for Joint Video Super-Resolution and Deblurring. Proceedings of the IEEE/CVF Conference on Computer Vision and Pattern Recognition.

[45] Zhou S., Li C., Chan K.C.K., Loy C.C. ProPainter: Improving Propagation and Transformer for Video Inpainting. Proceedings of the IEEE/CVF International Conference on Computer Vision.

[46] Liang J., Cao J., Fan Y., Zhang K., Ranjan R., Li Y., Timofte R., Van Gool L. (2024). VRT: A Video Restoration Transformer. IEEE Trans. Image Process..

[47] Hu E.J., Shen Y., Wallis P., Allen-Zhu Z., Li Y., Wang S., Wang L., Chen W. LoRA: Low-Rank Adaptation of Large Language Models. Proceedings of the International Conference on Learning Representations (ICLR).

[48] Jia M., Tang L., Chen B.C., Cardie C., Belongie S., Hariharan B., Lim S.N. Visual Prompt Tuning. Proceedings of the European Conference on Computer Vision.

[49] Chen S., Ge C., Tong Z., Wang J., Song Y., Wang J., Luo P. (2022). AdaptFormer: Adapting Vision Transformers for Scalable Visual Recognition. Advances in Neural Information Processing Systems.

[50] Houlsby N., Giurgiu A., Jastrzebski S., Morrone B., de Laroussilhe Q., Gesmundo A., Attariyan M., Gelly S. Parameter-Efficient Transfer Learning for NLP. Proceedings of the 36th International Conference on Machine Learning.

